# Health literacy: health professionals’ understandings and their perceptions of barriers that Indigenous patients encounter

**DOI:** 10.1186/s12913-014-0614-1

**Published:** 2014-11-29

**Authors:** Michelle Lambert, Joanne Luke, Bernice Downey, Sue Crengle, Margaret Kelaher, Susan Reid, Janet Smylie

**Affiliations:** National Institute of Health Innovation, School of Population Health, University of Auckland, Auckland, New Zealand; The Victorian Aboriginal Health Service, Fitzroy, Melbourne, Australia; Doctoral Candidate, McMaster University, Centre for Research on Inner City Health, Li Ka Shing Knowledge Institute, Saint Michael’s Hospital, Toronto, Canada; Centre for Health Policy, Programs & Economics, Melbourne School of Population and Global Health, University of Melbourne, Melbourne, Australia; Workbase Education Trust, Ponsonby, Auckland, New Zealand; Centre For Research on Inner City Health, Li Ka Shing Ka Shing Knowledge Institute, Saint Michael’s Hospital, Toronto, Canada; Dalla Lana School of Public Health, University of Toronto, Toronto, Canada

**Keywords:** Indigenous health, Health literacy, Cardiovascular diseases, Medication knowledge, Community based participatory research

## Abstract

**Background:**

Despite the growing interest in health literacy, little research has been done around health professionals’ knowledge of health literacy or understandings of the barriers to health literacy that patients face when navigating the health care system. Indigenous peoples in New Zealand (NZ), Canada and Australia experience numerous inequalities in health status and outcomes and international evidence reveals that Indigenous, minority, and socio-economically disadvantaged populations have greater literacy needs. To address concerns in Indigenous health literacy, a two-pronged approach inclusive of both education of health professionals, and structural reform reducing demands the system places on Indigenous patients, are important steps towards reducing these inequalities.

**Methods:**

Four Indigenous health care services were involved in the study. Interviews and one focus group were employed to explore the experiences of health professionals working with patients who had experienced cardiovascular disease (CVD) and were taking medications to prevent future events. A thematic analysis was completed and these insights were used in the development of an intervention that was tested as phase two of the study.

**Results:**

Analysis of the data identified ten common themes. This paper concentrates on health professionals’ understanding of health literacy and perceptions of barriers that their patients face when accessing healthcare. Health professionals’ concepts of health literacy varied and were associated with their perceptions of the barriers that their patients face when attempting to build health literacy skills. These concepts ranged from definitions of health literacy that were focussed on patient deficit to broader definitions that focussed on both patients and the health system. All participants identified a combination of cultural, social and systemic barriers as impediments to their Indigenous patients improving their health literacy knowledge and practices.

**Conclusions:**

This study suggests that health professionals have a limited understanding of health literacy and of the consequences of low health literacy for their Indigenous patients. This lack of understanding combined with the perceived barriers to improving health literacy limit health professionals’ ability to improve their Indigenous patients’ health literacy skills and may limit patients’ capacity to improve understanding of their illness and instructions on how to manage their health condition/s.

## Background

Recently there has been increased research interest in the field of health literacy and the impact of literacy on the patient-health professional relationship [1-11]. However, there has been little research about health professionals’ understandings of health literacy [12-14] and of the barriers that face their patients who have greater health literacy demands [10,15-18].

Health literacy is a widely used term that encompasses a range of ideas and definitions. Each of the countries involved in this study have official definitions: New Zealand (NZ) defines health literacy as “the ability to obtain, process, and understand basic health information and services to make appropriate health decisions” [19]. Australia uses the definition, “the knowledge and skills needed to understand and use information relating to health issues such as drugs and alcohol, disease prevention and treatment, safety and accident prevention, first aid, emergencies and staying healthy” [20], and a report by the Canadian Expert Panel on Health uses the definition: “The ability to access, understand, evaluate and communicate information as a way to promote, maintain and improve health in a variety of settings across the life- course” [21].

Each of these definitions implies that health professionals, healthcare providers, and the health system will play a major role in assisting people to build knowledge and skills about their health.

People with adequate levels of health literacy have the skills to seek out, critically read, interpret and understand health messages, treatment options and health professionals’ instructions. This in turn can lead to improved management of long term and chronic conditions (e.g. diabetes, CVD), increased use of preventative services (such as screening) and decreased use of emergency services [1,19,22,23]. In contrast, patients who have high health literacy demands placed on them (for example initial diagnosis of CVD) will have lower levels of health literacy knowledge and skills and if not supported through their encounters with health professionals and systems, (particularly for patients with complex medication regimes) will have limited opportunities to build their health literacy skills leading to unfavourable health outcomes [7,16].

Indigenous peoples in NZ, Canada and Australia experience numerous inequalities in health status and outcomes compared to their non-Indigenous peers [24] and international evidence reveals that Indigenous, minority, and socio-economically disadvantaged populations all have greater literacy needs [16,19,25]. In New Zealand the 2006 Adult Literacy and Life Skills Survey reported that 44% of adults surveyed had inadequate literacy [26]; data from the same study revealed 65% of Indigenous Maori adults had inadequate literacy [26]. Furthermore, levels of health literacy are poor with more than 56% of the NZ adult population having inadequate levels of health literacy to meet their basic health needs. A higher proportion of Maori males (80%) and females (75%) have poor health literacy than their non-Maori peers [19].

In Canada, the 2003 International Adult Literacy and Life Skills Survey (IALLSS) reported that 48% of adults surveyed had inadequate literacy [21]. Data on Aboriginal adult literacy in Canada is scarce. The Canadian Council on Learning (CCL) reports the proportion of Aboriginal adults with inadequate literacy scores is at least 16 percent higher than their non-Aboriginal counterparts in urban Manitoba and Saskatchewan, the Yukon, Northwest Territories and Nunavut [21]. In terms of health literacy, an estimated 55% of the Canadian adult population had an inadequate score on the IALSS health literacy scale.

In Australia, the 2006 Adult Literacy and Life Skills Survey (ALLS) reported that 46.4% of adults surveyed had inadequate literacy [27]. Like Canada, there is a lack of comprehensive information on levels of literacy for Indigenous peoples but an Australian Bureau of Statistics report [28] indicated that 41% of Aboriginal people had very low literacy. In terms of health literacy, an estimated 60% of the Australian adult population had an inadequate score on the ALLS health literacy scale.

### The evolution of health literacy

Health literacy is an evolving concept developed in the United States (USA) in the 1970s [29]. Health literacy was initially framed as a patient deficit issue where low health literacy was considered a ‘risk’ in a healthcare system [7]. In this construction, low health literacy is considered a patient characteristic, influenced by factors such as the patient’s level of reading and education. Interventions to improve health literacy needed to focus on the patient. This early definition did not consider influences on health literacy outside of personal patient attributes such as the role of healthcare providers, communication by and with health professionals, and attributes of the health services and systems that patients have to navigate.

In the past two decades, the concept of health literacy has evolved considerably. In the 1990s the role of health professionals in building or limiting patients’ health literacy was highlighted for the first time [7]. The role health professionals play in communicating effectively and in supporting the development of patient’s health literacy continued to receive attention in the 2000s [10,30]. In 2004, an Institute of Medicine report [7] suggested health services and systems also placed significant health literacy demands on patients through various factors - including the services’ and systems’ design, operational activities and built environment [31]. From here a new understanding emerged that patients and health professionals cannot address health literacy needs independently; healthcare services and health systems need to support health professionals to build patient health literacy by reducing the demands placed on patients, and by supporting health professionals to communicate more effectively with patients [32].

To address concerns in Indigenous health literacy, a two-pronged approach that is inclusive of both health professionals building health literacy among Indigenous populations, and structural reform to reduce demands the system places on Indigenous patients, are important steps towards reducing these inequalities [7,19].

In this paper we explore the understanding and perceptions of health professionals who work with Indigenous patients and address the following questions:

1. what are their understandings of health literacy
2. what are their perceptions of the barriers facing Indigenous people when attempting to build better health and
3. what strategies do they use to build patient health literacy in their day to day practice

## Methods

This paper reports findings from phase one of an international Indigenous health research project that included NZ, Australian and Canadian participants. The overall goal of the project was to develop and trial an intervention to improve health literacy relating to medicines used to prevent cardiovascular disease (CVD). Interviews were conducted with health professionals who work with patients who had experienced CVD and were taking medications to prevent future events. The in-depth information yielded from these discussions about the experiences, perceptions and practices of the health professionals in relation to their patient’s health literacy skills and CVD medication knowledge will be used to ensure the appropriateness of any intervention developed.

### Setting

Four Indigenous comprehensive primary health care services were involved in the study: an urban and a rural Maori health service in NZ, an urban Aboriginal health service in Australia, and an urban Indigenous health service in Canada.

Ethics approval was obtained from the Health and Disability Multi-region Ethics Committee (MEC/10/061/EXP) in NZ; the University of Melbourne Research Ethics Committee (1238349.4) in Australia; and the St Michael’s Hospital Research Ethics Board (10–324) in Canada.

### Data collection

At each site, potential participants from a range of roles (nurses, doctors, service managers, community health workers, pharmacists, and receptionists) were purposively selected and invited to participate. All participants approached at each site accepted the invitation to be involved. After obtaining informed consent, all participants were interviewed individually or as part of a focus group. The interviews were in-depth, semi-structured and lasted between 40 and 60 minutes. One or two Indigenous researchers conducted the individual interviews and the focus group in private rooms at the healthcare centres where the staff were employed. Participants anonomity and confidentially was ensured by using unique identifers for all participants when transcribing and analysising all data.

An interview schedule was collaboratively developed and adapted to guide interviews in all three countries. This included probing for information on: the participants’ awareness and understanding of health literacy; opinions about the environment in the health service with respect to suitability for people with health literacy needs; the health literacy needs they observed among patients and how these needs affected patients’ health and the health professionals’ engagement with patients; what strategies the professionals used to build health literacy skills and knowledge to meet patients’ health literacy needs; and professionals’ opinions on how health literacy problems could best be addressed. The guide ensured that all countries canvassed important aspects of the topic while allowing flexibility in how the interview was conducted and the inclusion of any discussion points that were country-specific. Detailed notes were taken during the interviews. With one exception, interviews were also audio recorded and transcribed verbatim.

Twenty-four individual interviews and one focus group were completed. The focus group involved five staff members including a doctor, a service manager, a receptionist and two nurses. Participant characteristics are described in Table 1.

**Table 1:** **Participants demographics**

**Demographics**		**New Zealand**	**Australia**	**Canada**
Gender (n = 23)	Female	12	4	
	Male	4	3	
Ethnicity (n = 29)	Indigenous	2	2	0
	Non- Indigenous	13	5	6
Role (n = 29)	Doctor	4	2	1
	Nurse	7	1	3
	Community Health worker	1	2	0
	Specialist staff (Pharmacist, Diabetes educator)	0	2	2
	Service Manager	2	0	0
	Receptionist	1	0	0
Site (n = 29)	Urban	6	7	6
	Rural	10	0	0

### Data analysis

A thematic analysis was completed by the research teams within each country. This was undertaken in three steps: first, the research team reviewed their country’s interview transcripts to familiarise themselves with their data and to facilitate discussion about the themes; secondly, the researchers created a thematic framework to chart that data (based on the research questions and recurrent issues identified from participants’ accounts); and finally, researchers independently coded the transcripts and met regularly to review and refine the coding framework to ensure it covered the most important findings from the data. One researcher from each country then met (via Skype) to discuss results and compile a list of common themes from all three countries. The NZ team used Nvivo 9 to assist with data management and two matrix coding analyses were conducted to draw out the differences between demographic groups – matrix analysis by role and gender.

## Results

Ten themes and corresponding sub themes were found to be common to all three countries. (see Table 2).

**Table 2:** **The common themes identified by each country**

**Main themes**	**Sub themes**
1. Health professionals role in health service	● What role involves
	● Boundaries of the role
	● Patient expectation of their role
	● Expectations of other health professionals role
2. Health professionals’ expectations of patients	
3. Relationship with community	
4. System/service issues	
5. Understanding of health literacy*	● Patient
	● System
6. Assessing health literacy	● Communication
	● Encourage/acknowledge health literacy practices
	● Identify patients health literacy needs
	● Building health literacy
7. Perceived barriers to health literacy*	● Systematic/structural
	● Social
	● Cultural
8. Working with other health professionals	● Doctors
	● Nurses
	● Other health professionals
9. Health literacy resources	● Pamphlets
	● Visuals
	● Videos
10. Effective strategies for working with low health literate patients	● Support role/advocate
	● Tailored care
	● Communication
	● Reinforcement
	● Engagement with patients

*These themes are the focus of this paper.

Theme strings 5 (health professionals understanding of health literacy) and 7 (health literacy barriers that their patients face when accessing healthcare) are the focus of this paper.

### Varying levels of understanding of health literacy among health professionals

The health professionals had varying levels of understanding of health literacy. Across the three countries, many health professionals were not familiar with the term ‘health literacy’, and discussed the concept in a manner most closely aligned with a patient deficit focus. As such, health literacy was conceptualised at the ‘patient’ level and centred on patients’ individual skills such as reading, writing, understanding, and navigating. This understanding was described by health professionals across all three countries.

“Health literacy is an ability of a person really to look at information regarding health, find it, access it and once they have that information, read it, observe it and interpret it. And that information can be from a book, a piece of paper, a television show, from the doctor, from a variety of sources. And then once they get that information it’s kind of to do something with it like, for instance, look at a food label and say this is healthy or not healthy, maybe judge on it and act on it.” (Nurse, Canada)

Health professionals also viewed the patient’s ability to navigate healthcare services and the health system as a large component of health literacy. One participant described health literacy as being

“… about icons and getting their travel to hospital and having an understanding of hospitals… you know kind of navigation of a hospital…… but it is being explored a lot more…. expanded to include navigation of all (health) information.” (Service Manager, NZ)

This notion of patients having navigation skills was reiterated by another participant.

“But my understanding [health literacy] is people having a sort of workable knowledge of how they interact with the health system.” (Doctor, Australia)

Another doctor expanded on this definition by adding medication knowledge.

“I almost look at it as an ability to navigate the system and also…. being able to access health. Which health professionals do certain jobs, but also an understanding about the medication and what it is used for and obviously compliance to medication.” (Doctor, NZ)

Other participants had a wider understanding of health literacy. One participant recognised that health professionals had a role in building patients health literacy.

“… have I made it clear to patients what their treatment is, what their condition and the risk factors [are] and what they should expect to happen to them if we’re on the right track. Where they can go if they need to ask questions about it and that type of thing. And you know all the resources that are there.” (Health Professional, Canada)

A description like this moves some of the responsibility to the health professional, the heath care provider and the health system. Another health professional described health literacy as

“a really key part of people being able to, you know, go to a hospital appointment and not find it really stressful or traumatic or knowing when to present with things.” (Doctor, Australia)

Although these descriptions include more of an interface between the patient, the health care provider and the health system, there is still an element of patient responsibility.

There were differences in how different groups of health professionals conceptualised health literacy in NZ. Doctors were more likely than other health professionals to see lack of health literacy as a deficit in patients:

“My interpretation of what health literacy means is what (is) the understanding of the individual about health in general, and particularly about their own health.” (Doctor, NZ)

In contrast, nurses and community health worker participants were more likely to see health literacy as related to communication between health professionals and patient’s.

“Health literacy has got nothing to do with somebody’s understanding of the words and it’s got a lot to do with who the messenger is and the relationship of the messenger to the person you want to get the message. It has got a lot to do with the tone and the feeling and the emotions around that relationship.” (Nurse, NZ)

This conceptualisation is shifting more of the responsibility to the health professional, provider and system. Interestingly, this distinction between health professionals was not present in the Australian interviews, where the two longstanding doctors who had worked in Aboriginal health for many years had a progressive understanding of health literacy that extended beyond the patient.

### Perceived barriers to improving patients’ health literacy linked to health care professionals’ understandings of health literacy

During the analysis of the interview transcripts it became clear that health professionals’ perceptions of the barriers to improving a patient’s health literacy were closely aligned to the participants’ understanding of health literacy. Many barriers were identified during the analysis of the interview transcripts and these were grouped into general categories: cultural barriers, social barriers and structural barriers.

### Non-indigenous biomedical primary health service approaches present an overarching cultural barrier to understanding

Staff across the three countries were employed in medical services modeling a biomedical primary health care service. This was seen by some participants as an overarching cultural barrier, as medical language and health concepts used within these services derive from the dominant culture and often compete with Indigenous perspectives and understandings of health. This has a large impact on both patient and health professional understanding and communication.

Difficulties in or, in some cases, exclusion from Western education for Indigenous people has meant that many health professionals identified a patient’s lack of Western education as a barrier to building health literacy.

“So the signage, Yeah but I guess if people can’t read then that could be a bit of a challenge.” (Health Professional, Australia)

“When does health programming start in school? ....I hope the word is mandatory, but mandatory public education. Depending on what one learns and is taught there and what one engages with there. I mean, that shapes one’s consideration for health you know, for a lifetime, probably and the norms of the family.” (Health Professional, Canada)

Another participant reflected on how limited Western education leading to unfamiliarity with biomedical concepts leads to problems understanding health information.

“Yeah, lack of a good education I have encountered now and again where people go, ‘So smoking [marijuana] is bad but smoking cigarettes is alright.’ …People can go through their whole life and they just haven’t got the basic information that you know smoking is stuffing your lungs up whatever you are smoking.” (Health Professional, Australia)

A few health professionals spoke of patients lacking “basic” knowledge or “basic” information or being unfamiliar with medical jargon.

“I’ve encountered clients who…almost verbatim, I was asked how low is too low for a blood sugar reading. And....that’s very basic information.” (Health Professional, Canada)

Many health professionals interviewed, acknowledged that the patient-health professional relationship operates from a position of power, where health professionals communicate their expertise.

“Sometimes I ask, “you just saw the doctor, why didn’t you ask the doctor?” And they say, “the doctor is in a hurry, there’s lots of patients in there, and they haven’t got enough time to do that, talking about that.” (Nurse, NZ)

### Multiple social barriers can prevent uptake of information

Many Indigenous patients face multiple social issues that are often overwhelming and compete with health issues for attention. Poverty related to low income, for example, may be seen as a more pressing priority than a health issue. A doctor from Australia summarised this idea

“ their transport doesn’t work or they have got major other responsibilities in their life such as funerals, or child care or elderly people to look after or drug issues or abusive domestic violence situations then they are going to miss out on that information.”

Not only can social factors interfere with a patient’s ability to absorb or understand health information, they can also inhibit patients turning knowledge into action. Social factors such as low income or geographic isolation can affect this knowledge activation. A Canadian nurse discussed this

“Sometimes you have to dig further and see if there’s more issues involved with the situation that are inhibiting them. It could be financial. It could be mental health; their family situations, living situations. It’s not as easy for some as it is for others but I think within this culture itself too, it can be very difficult for some, is what I’m learning.” (Nurse, Canada)

Another competing social barrier is that Indigenous health is largely problematised and described in deficit by the dominant culture [33]. As one participant commented

“It’s almost normality that you would drink. You will smoke and you will have other vices; and your health will go to hell”. (Nurse, NZ)

This inevitably of poor health was seen as an important barrier to building patients’ health literacy as health professionals were contending with the fatalistic beliefs of patients who perceived they had limited control over their health and as a result normalise poor health.

### Service delivery and design can help or hinder

In the context of Indigenous health, lack of safe and affordable access to services is an overarching barrier to improving health literacy [33,34]. A large part of the discussion about access was around navigation and how the built environment is set up to facilitate health literacy. Facilities such as the onsite pharmacy at the Australian site, were seen as a strong facilitator to improving health literacy.

“Oh look, having our own pharmacy is a great help. Yeah it is a great help, otherwise a lot get lost along the way. Even though the [mainstream Pharmacy] is just around the corner a lot of people get lost along the way…so having our own pharmacy is just great.” (Health Professional, Australia)

One participant in NZ reflected on how the common service delivery model is not always conducive to building health literacy.

“The setup of a normal GP practice is to get them in and get them out as quickly as possible. And I’m sure that they must pick up on that, they must do. So the atmosphere is all wrong for asking questions, and talking around the subject, and things like that.” (Nurse, NZ)

“I think people have a hard time with health literacy and I think that we do less to support their inherent characteristics just simply because of time and resource constraints. … and then to motivate them, it is very clinician dependent so whether that health system is doing them justice… I think certainly with the aboriginal population there needs to be more things that are culturally sensitive and that they can identify with.” (Health Professional, Canada)

Similarly in Australia and Canada, the notion that health professionals need that time to build health literacy was expressed.

“Time is an issue, especially now because we’re in transition too. Yeah, time is a big issue.” (Health Professional, Canada)

“… the conversations that really build and support health literacy tend to fall away when there is long waiting lists, you know and there are people in the waiting room and it is chaotic.” (Doctor, Australia)

An integral component of health literacy is the communication between patients and health professionals. Many of the participants understood the importance of communication but also discussed the barriers to good communication. One participant in Canada stated

“I think there is a benefit to building rapport and building trust and building a sense of communication between the health care provider and the client.” (Health Professional, Canada)

Good communication can lead to better patient understanding and patient adherence. This was highlighted by a nurse

“you must take it [medication], you know and then you have that conversation with them. And once they’ve explained the importance of taking it, and what the actual medication does, they’re quite happy to take it.” (Nurse, NZ)

But it is also important to note that health information can be both complex and unfamiliar and good communication can take more than one session to ensure the patient builds the required knowledge and skills.

“The more times people get the message, eventually they take away a little bit each time. And eventually over a period of time, hopefully they put all the little pieces together.” (Nurse, Australia)

Patients can often be confused by the language health professionals use when they are talking with patients. The use of unfamiliar medical terms can cause confusion for the patient at best or can scare the patient away. They may quickly end the session to avoid embarrassment of having to ask ‘dumb’ questions. A NZ doctor describes this

“We can’t talk in high faluting English… break it down to issues that are important to them. Goals that are important to them.”

“I think it is important not to use really big terms with patients like sometimes the medical words can be really kind of confusing for people. So I think putting things into plain language makes things a lot easier for people to understand.” (Nurse, NZ)

Participants recognized that patients and health professionals were not able to fully build health literacy without adequate sources of information. In the context of medications, one doctor acknowledged that access to good and impartial information was often complicated by overarching systemic barriers where pharmaceutical companies controlled what is available.

“That is not very useful sources of information because it comes through the drug companies and they are more interested in litigation than they are in patient information.” (Doctor, Australia)

When considering the resources to build health literacy, participants felt it important that resources were pitched at the right level.

“They see me coming with my arms full of pamphlets and honestly I think they go straight into light the fire [but] you can get away with it once and so I’ve been very selective about what I think would suit that person or that family. Because you sort of get one shot at it with the brochures.” (Nurse, NZ)

Many other staff across the three countries also observed that for Indigenous populations resources needed to be culturally accessible.

“Yeah, ‘cause sometimes we get resources but they are kind of from other areas and they might not be appropriate to the local community.” (Health Professional, Australia)

Participants spoke of service level issues and the realities of Indigenous health services. In NZ, a lack of consistency in care due to turnover of staff was noted as a barrier to patients’ developing their health literacy.

“There’s this uncertainty and he gets really confused about the change of pills and different doctors and who said this and who said that.” (Nurse, NZ)

Another issue identified, was that health services are geared towards acute problem management and have higher turnover of staff.

“When we’re short staffed it puts people under pressure and you tend to have to prioritise things so you tend to do the more urgent stuff and the conversations that really build and support health literacy tend to fall away.” (Doctor, Australia).

This was reiterated in NZ, where patients experience a lack of consistency in the relationship due to high staff turnover. This can affect the patients’ willingness to engage with both the health professional and as a consequence their health condition.

“We have quite a, you know turnover of GPs. A lot of locums…….they [patients] will say yes, yes, and walk away with no real understanding of what’s going on with them, and why they’re taking their medication.” (Nurse, NZ)

Various staff across the three countries acknowledged that, historically, Western health services have limited Indigenous people’s access to health services and that their services had a role in creating safe accessible spaces for Indigenous peoples. These staff understood that services have a role in reducing the demands placed on patients and health professionals. One nurse reflected how access was facilitated through the creation of a community space.

“We are such a community centre. That we all, you know people communicate there a lot; laugh, joke, feel relaxed and when people are relaxed they take stuff in. When people are stressed, tense and unsure, nothing goes through; you know they have got all these barriers up. But if people are relaxed and feeling at ease, then that is when they learn and I think really engenders that well.” (Nurse, Australia).

“If you are going to do it right - there should be an organisational decision to provide the resources and the time and to formalize the strategies or the mechanisms that you are going to put in place, so that everybody’s doing it ....and they are doing it properly and in the same way.” (Nurse, Canada)

## Discussion

The majority of health professionals were unfamiliar with the term health literacy and believed it had to do with patients’ skill at managing their health and navigating the health care system. Their understandings of the barriers that restrict patients from developing better health literacy skills were closely linked to their patient focused understanding of health literacy as a whole. However, some participants discussed, to some extent, cultural, social and systemic or health system barriers that they believed impeded their patients’ developing better health literacy skills.

Our study expands the evidence that many health professionals have limited understanding of health literacy. Furthermore, the vast majority of participants had a limited understanding of the role that the healthcare system and health professionals play in building patients’ health literacy skills [14,35]. Both these findings build on research done by Devraj & Gupchup, [33] and Cafiero [12]. Devraj & Gupchup found low levels of understanding of health literacy and the consequences of poor health literacy among 701 Illinois pharmacists. Cafiero found limited knowledge of health literacy and health literacy strategies among nurse practitioners who worked in outpatient settings.

Health professionals’ understandings of the barriers that patients face in developing health literacy skills have not been widely studied. This lack of understanding as well as health provider related barriers, such as lack of time and the availability of convenient delivery mechanisms, were reported most frequently by previous studies [33,34]. Our study, which includes a more diverse group of health professionals, confirms many of the systemic barriers highlighted by these previous studies and identifies other perceived barriers including the cultural and social barriers that many Indigenous patients face when attempting to develop their health literacy skills. Cultural barriers such as a concentration on western health concepts, lack of Indigenous health professionals as well as social factors, such as poverty that can overwhelm patients and impact on their ability to prioritise health, are barriers that health professionals interviewed identified as important to address in any intervention to increase health literacy. An additional systemic barrier identified by health professionals in this current study was the service delivery model (patient in, patient out) which was not seen as conducive to building health literacy skills.

The results of this study also raise more questions about the best solutions to building patient health literacy - whether interventions focusing on patient education; health professionals’ training; or reducing system demands or a combination would yield the most improvement in patient understanding of illness prevention, diagnoses, medications and instructions on how to take care of their health condition/s.

### Strengths and limitations

This study was conducted in three countries and included staff from four Indigenous health services. Participants included a variety of health care staff allowing for more breadth of knowledge of various areas of health care rather than just a select group, (for example pharmacists or nurses), as earlier research has concentrated on. Although 29 health professionals were interviewed in total, the number is still relatively small and the findings may not be generalisable across all health professionals. Furthermore, the health professionals interviewed were primarily working with Indigenous peoples and the findings may not reflect the understandings of health professionals who work with non-Indigenous peoples. In spite of this some barriers identified may not be unique to indigenous populations, for example the understanding of biomedical concepts is likely to be an issue for a considerable proportion of most populations.

## Conclusions

This study suggests that health professionals have a limited knowledge both of health literacy and of the consequences of low health literacy for their Indigenous patients. The findings from this qualitative analysis have been applied in the development of a customized, structured CVD medication programme that is delivered by health professionals, to Indigenous people with, or at risk, of CVD. We have undertaken a clinical trial of the programme in phase two of the project.

The goal of interventions to improve health literacy is to improve patient health outcomes by ensuring patient knowledge and skills of all important aspects of their condition and treatment. This can be done by clinician-patient interventions which include effective communication techniques, for example patient-centered communication and confirmation of new knowledge and skills. . Yet, research has shown that when attempting to improve the health literacy of patients, health professionals can be limited by barriers within the healthcare that employs them [36]. These barriers are often the result of the health system in which the healthcare service and health professional operates. Consistent with our findings the literature shows interventions need to look at ways healthcare services and health systems can support health professionals, patients and families to develop enhanced and effective health literacy as well as how demands on patients can be reduced [37,38].

Other recommendations from this initial phase include: providing health literacy training for health professionals; development of accessible information tailored to low health literate patients and their particular condition; and minimising system barriers, for example time restraints and over medicialised language.

Training is an important way to address low health literacy awareness and understanding among health professionals. This training should cover basic information and theory about health literacy, a Universal Precautions approach to health literacy [39], and strategies to use to build health literacy among patients.
